# Plants Inspired Biomimetics Architecture in Modern Buildings: A Review of Form, Function and Energy

**DOI:** 10.3390/biomimetics10020124

**Published:** 2025-02-19

**Authors:** Maryam Bijari, Ardalan Aflaki, Masoud Esfandiari

**Affiliations:** 1 Faculty of Architecture and Art, University of Guilan, Rasht 41996-13776, Iran; 2 SYSTRA S.p.A, 38123 Trento, Italy

**Keywords:** adoptable building façade, biomimicry architecture, daylighting, energy-efficient building, nature-based solution

## Abstract

Biomimicry architecture provides innovative solutions to contemporary environmental challenges by drawing inspiration from nature’s strategies to enhance sustainability and energy efficiency in the built environment. Plants, with their remarkable ability to adapt to changes in light, temperature, and humidity, serve as a central model for biomimetic design due to their potential to optimize energy use and improve building performance. By leveraging these natural principles, biomimetic architecture can significantly reduce carbon emissions and create eco-friendly structures that respond dynamically to environmental conditions. This approach not only addresses the urgent need for sustainable development but also fosters harmony between human-made environments and the natural world. This study offers a comprehensive review of biomimetic technologies, focusing on their role in improving energy efficiency and building performance. Also, it examines a range of global case studies that have successfully implemented biomimicry, showcasing its versatility and effectiveness across diverse environmental and architectural contexts. Based on these insights, this research proposes a novel design inspired by the moonflower plant, which adapts to its environment by responding to external stimuli. The proposed design applies these adaptive strategies to architectural solutions, enabling buildings to optimize performance by dynamically interacting with environmental conditions such as light and temperature. By analyzing biomimetic principles and their applications, this study contributes to the growing body of knowledge on sustainable architecture. It highlights the potential of biomimicry to balance environmental sustainability with economic growth, offering valuable insights for architects, designers, and policymakers seeking to create greener, more efficient built environments.

## 1. Introduction

The increasing impact of global climate change has sparked worries about environmental harm, and the release of greenhouse gases from these activities leads to irreversible damage [1,2]. Consequently, cutting down on greenhouse gas emissions has been a key focus in recent years to curb the rise in global temperatures [3]. One important consideration for reducing energy consumption and minimizing the carbon footprint is to manage the use of electricity and artificial lighting in buildings. Artificial lighting typically requires significant energy, often sourced from non-renewable resources such as oil [4]. By controlling consumption, artificial lighting not only can improve the quality of the environment and reduce psychological and physiological issues in humans, but also helps to reduce the use of non-renewable energy sources and minimize the undesirable impacts on the environment [5].

Nature provides an abundant supply of solutions for numerous scientific and technical challenges. Over billions of years, biological systems have evolved to create advantageous relationships between structure, properties, and performance that are essential for survival [6]. By emulating these natural systems, buildings can be designed to maximize natural light exposure, reduce glare and heat gain, and utilize dynamic shading systems that adapt to changing conditions [7,8].

Biomimicry, as a scientific field, involves an interdisciplinary approach and has the capacity to offer sustainable solutions through the collaboration of biologists, physicists, chemists, engineers, and architects [9]. The importance of this approach in architecture has increased because biomimicry brings several inspirations from nature and introduces great potential to create a sustainable, energy-efficient built environment. This great opportunity is more tangible, particularly today, because new building materials and new construction techniques can be seen more than ever in the past [10,11]. On the other hand, plants utilize various mechanisms to harness sunlight efficiently, making them a prime model to emulate for sustainable structures that maximize natural light utilization [12].

Despite the studies and activities carried out in this field, there are still many unresolved problems and issues that require more attention. One of these issues is designing biomimicry architecture, which requires the development of cognitive systems that support the early stages of the innovation process, including problem-based information retrieval and formation and estimation. So, this article aims to examine the implementation of biomimicry with a focus on inspiration from plants in a built environment to reduce the use of non-renewable energy resources and fossil fuels for lighting. It assesses the effectiveness of this approach by looking at various aspects, such as maximizing natural light and decreasing the use of artificial lighting. Ultimately, it seeks to improve and address existing challenges in this area with creative and efficient solutions, taking a step forward toward employing biomimicry architecture to reduce environmental pollution and carbon footprint.

## 2. A Literature Review

### 2.1. Biomimicry

Contreras Lezcano [13] is known as the founder of the biomimicry movement. She set out all her theories in the book “*Biomimicry: Innovations Inspired by Nature*”. A year later, together with Dayna Baumeister, they founded biomimicry, which puts into practice a deep understanding of the biological adaptation of organisms to help architects, engineers, and designers solve design and engineering problems in a sustainable way. Biomimicry is a relatively new scientific field that involves studying nature’s designs and mimicking them to solve human problems. Essentially, designers look at nature as a “model, measure, and mentor”, with one of its key goals being sustainability and environmental preservation [13].

The term biomimicry comes from the Greek word “bios”, meaning life, and mimesis, meaning imitation. Biomimicry is defined as “the abstract design of good ideas from nature” or “emerging disciplines that mimic designs and processes of nature to create healthier and more sustainable planets” [14]. The idea behind this concept is to emphasize sustainability as a main goal of biomimicry, which, when applied to building design in order to increase the strength of materials through self-healing and self-assembly properties, can provide better solutions to increase building performance, save energy, and provide material cost savings by eliminating waste [15].

Biomimicry is all about reimagining humans and about what to do to make industrial life possible. This perspective introduces nature as a mentor to walk alongside and respect it [16]. Biomimicry is a brilliant approach to looking for sustainable solutions to human problems by mimicking and emulating nature in its analogies, phenomena, and patterns. Biomimicry’s main aim is to make a great design by mimicking the different living organisms, which have been evolving for 3.8 billion years [17]. Biomimicry imitates living biological systems in non-living systems based on the characteristics of a living biological system [18]. In architecture, biomimicry manifests as both a tool and ideology, leading to holistic designs that reflect biological processes [19].

### 2.2. Biomimicry in Architecture

In recent years, a nature-inspired approach to architecture has emerged as a way to mitigate the negative impacts of the industrial age, enhancing urban living while creating visually appealing super-tall buildings [20,21]. In fact, biomimicry facilitates the expansion of sustainable thinking through principles such as connectivity and system integration. In architecture, it is an interdisciplinary design approach that has not yet been fully explored, but it can expand the designer’s realm of ideas across various fields [22]. While nature is imitated, it is not merely reproduced; instead, it requires further abstraction to derive solutions inspired by natural models. These solutions align with life’s principles, potentially reducing environmental burdens by being less reliant on fossil fuels and more self-organizing and multifunctional [23,24].

Depending on the usage, biomimicry in the literature has various definitions. In sustainability, it refers to mimicking biological forms, processes, and systems to develop sustainable solutions. Ecosystem design strategies involve applying lessons from the natural world to support sustainable architecture and urban planning. Furthermore, design takes an interdisciplinary approach, studying and adapting concepts from nature to address design challenges [25].

**Figure 1:**
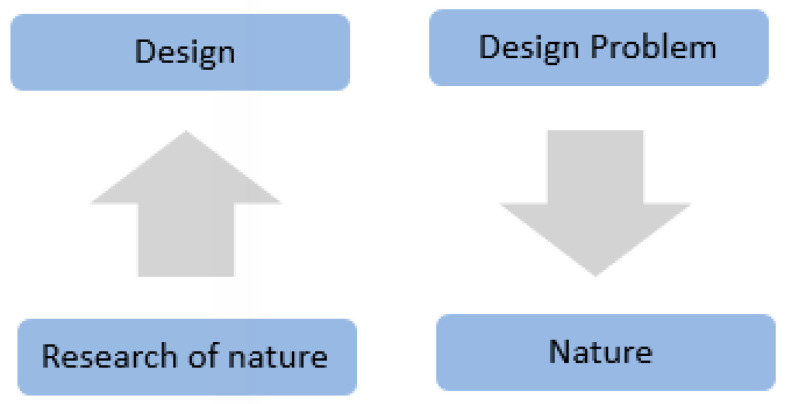
Biomimicry approaches.

As shown in Figure 1, biomimicry approaches are usually divided into two categories: defining human design needs or problems and searching for solutions from other organisms or ecosystems to solve this problem or designing based on biology or identifying a specific feature, behavior, or function in an organism or ecosystem and translating it into human designs. This utilization of nature and living organisms can be used in three methods: copy, abstraction, and inspiration [26,27].

The organism level requires the designer to become inspired by a specific organism, like a plant or an animal, while also analyzing how it functions, using the whole organism or a part of it [28]. The behavioral level involves mimicking the behavior of a living being in its environment to draw inspiration from a structure or a process in design [29]. The ecosystem level includes imitating how a living being interacts with its environment and the number of its constituent elements, which are examined on a broader scale [27].

**Figure 2:**
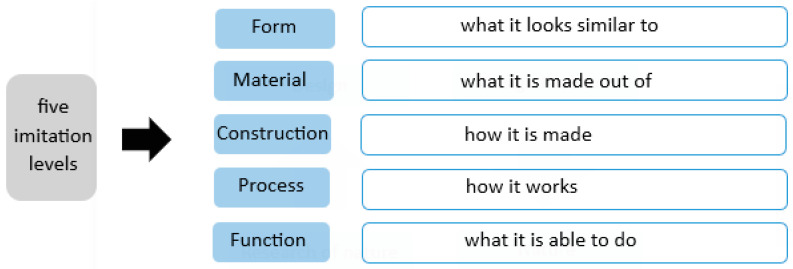
Level of biomimicry.

Biomimicry architecture has three levels of imitation, including imitation of organisms, behavior, and ecosystems. On the organism level, buildings may imitate the characteristics of an individual organism. On the behavioral level, the design may be inspired by how the organism behaves or relates to its larger context [30]. On the ecosystem level, design may draw from the entire ecosystem of an organism and its surroundings. It emphasizes natural processes and cycles of the greater environment [31,32]. Three levels of biomimicry determine which aspect of ‘bio’ can be ‘mimicked’ and applied to a design problem: the organism, the behavior, or the ecosystem level repeated [30]. As shown in Figure 2, at each of these levels, there are five other possible dimensions for imitation, including form, material, structure, process, and function [33,34].

### 2.3. Plants in Biomimetic Architecture

Inspiration from plants is often seen in biomimicry architecture, which is motivated by nature [35]. Trees and plants are flexible structures that are sensitive to climatic conditions, and as a response, they have developed a number of techniques and features that help them overcome such conditions [36]. Throughout history, there has always been a tendency to mimic natural forms of plants and incorporate their beauty into the designs. This has laid the foundations of botanical design. With advancements in natural sciences, simple imitation of plants has gradually transformed into a more detailed examination of their structure and function [37].

There is a design approach aimed at enhancing the mechanical, adaptive, and environmental performance of buildings by mimicking the morphology and structure of plants [38]. Plants, being a rich and extensive source of inspiration, are utilized in various ways based on studies conducted on nature-inspired structures. Some plants are used for natural ventilation, others for efficient lighting solutions, and some can even contribute to acoustic considerations.

While some plants are only considered for aesthetics in architecture, others can be inspired or used to significantly improve building performance and efficiency [39,40]. For instance, the ability of plants to dispose of light as an energy source and to reuse it in case of need is unparalleled [41], which inspires controlling entry for sunlight in buildings. Also, regulating internal temperature is another significant characteristic of buildings inspired by plants or flowers [39].

Plant biomimetic design research primarily focuses on the plant’s form, color, texture, function, and structure. This design philosophy emphasizes integrating knowledge from ergonomics, art, new materials, and energy efficiency into the design of buildings, products, and materials. This approach contributes to multiple goals, including enhancing design aesthetics and practicality, balancing technology and aesthetics, and harmonizing the delicate combination of tradition and modernity [42].

When drawing inspiration from the structure and function of plants, this process can be referred to as Phyto-mimetics. Plants and buildings exhibit similarities in how they interact with the environment. Due to their predominantly immobile nature, plants have to autonomously manage their temperature, exposure to light, and other essentials. Similarly, building architecture needs to integrate mechanisms for managing solar heat gain, air circulation, temperature regulation, and provision of natural light to cater to the occupants [43]. Plants, due to their immobility, have developed specific mechanisms to protect themselves from various environmental factors such as darkness, light, humidity, rainwater, fire, temperature, freezing, air movement, and air quality. These adaptations evolve over time and generations in response to the constantly changing environment [44].

### 2.4. Light in Biomimicry Architecture

Natural light in buildings is very important because not only does it have positive physical and mental effects on individuals, but it can also reduce energy consumption and help in creating sustainable buildings with minimal carbon footprint [45]. The use of natural light in architectural spaces has been very useful in various aspects such as energy efficiency, cost control, user health and well-being, and prevention of electricity [46,47]. Therefore, natural light should be the main source of illumination during the daytime. This light should create intimacy and provide a comfortable visual and thermal environment for residents [48].

Passive solar energy requires the placement and design of buildings in such a way that they interact positively with the environment and climate, and the biomimetic approach reinforces this goal [49]. Therefore, nature can be a helpful guide in utilizing solar light [50]. Biomimicry principles are modeled after nature’s ways. They harness solar energy efficiently, utilize resources and energy minimally, match forms to functions, and effectively recycle and repurpose materials [51].

Plants are drawn toward sunlight as an inspiring source for architects. This shows that plants are attracted to sources of light [52] or have specific mechanisms to allow light to enter their internal organs [17]. Biomimicry offers potential solutions and inspirations for architectural lighting design projects.

Studying plant adaptation to solar conditions inspires effective design strategies to improve building performance. Integrating solar technologies with biomimetic adaptive solutions can lead to sustainable design [53,54]. Most plants need sunlight to survive and maintain their energy. They use photosynthesis to convert solar radiation into storable energy. Plants adjust their orientation and inclination according to different weather conditions [55]. Some of them are also able to actively track the sun because of their light-sensitive organs [56].

Drawing inspiration from the process of harnessing light from plants in architecture includes various aspects such as light control, light transmission, light reflection, light passage, light concentration, etc. With the knowledge of biomimicry and the help of plants, one can respond to one or more of these aspects [57]. For example, the Zehra flower basket illuminates its internal space by passing light through its translucent body, or the silver Ragwort can reflect light to a great extent and create scattered and filtered light, with many plants having the ability to utilize natural light [58,59].

Minimizing shadows through the building is another concept widely used in lighting design. Plants not only have specific ways to absorb and store sunlight, but they also have different methods to manipulate and control light. This principle is mainly seen among plants with phyllotactic geometry, which often harnesses light in building lighting design. These projects used the Fibonacci law [53]. Also, techniques in façade design for shadow casting inspired by plants can be used to modify the incoming light to the building [60]. Therefore, by incorporating plants and their potential to utilize natural light, buildings can benefit from this renewable energy in various ways.

Many studies have been conducted in recent years on biomimicry and its impact on sustainability and energy conservation, but emphasis has not been placed on the role of plants in this approach. In a study, biomimetic research within the framework of sustainable development goals has been applied. The alignment of biomimicry with key sustainable development objectives has been approved because of its interdisciplinary nature and potential to provide solutions related to healthcare [61]. In another study, the role of bionic design in promoting sustainable development has been evaluated by analyzing its applications in morphological, functional, and material aspects. The results address considerably the current challenges of bionic design in terms of environmental sustainability [22]. In recent years, there have been few studies that focus on potential solutions in nature in order to establish daylighting in buildings. In line with the importance of the potential of plants to utilize natural light to reduce energy consumption, a study has focused on designing a responsive biomimetic kinetic system inspired by the functional and adaptive principles of Gazania flowers [62]. Additionally, another paper examines the use of biomimicry to improve daylight performance in office buildings in Cairo, Egypt. Through simulating and drawing inspiration from natural processes, designs have been proposed to enhance energy efficiency with an emphasis on natural light [63]. Apart from the application of biomimicry science in the built environment, studies have been conducted on the extensive applications of plants in the fields of biomimicry and biological inspiration and their role in developing sustainable solutions in medicine, material science, and environmental technology [64]. According to the previous literature, compatible biomimetic solutions and their efficiency in the built environment in terms of daylighting concern and energy consumption are questionable. Moreover, innovative solutions based on the current literature in order to provide natural lighting and energy saving have not been presented. These concepts will be presented after analysis of the current literature in order to be evaluated in future studies.

## 3. Research Methodology

The methodology in this article adopts the approach of “Scientific Methods and Rationale for Systematic Literature Review (SPAR-4-SLR)” developed by Paul and colleagues [65]. SLR is a rigorous and systematic type of exhaustive review of the already existing literature on a given topic. The primary purpose of SLR is to synthesize results from various studies, which then gives researchers an opportunity to draw wider and fuller conclusions about their topic of interest. This methodology involves several fundamental steps, which include locating relevant research, selecting studies that are appropriate, and systematically evaluating the quality as well as the significance of the findings reported in those studies. The application of the SLR method cuts across several fields and disciplines; this attests to its applicability in resolving a wide variety of research problems while adequately synthesizing results, hence contributing to an enhanced understanding of intricate subjects in different areas [66]. Therefore, this research analyzes significant research works from 2005 to 2024 in reputable databases, including Scopus, Science Direct, and Google Scholar. The SPAR-4-SLR protocol is chosen due to its precise methodological framework that includes clearly defined inclusion and exclusion criteria for selecting studies, helping to maintain high standards of evidence and ensuring that only the most relevant and reliable studies are included in the review.

As shown in Table 1, the review process is divided into three distinct stages. The first stage involves identifying and reviewing the relevant literature and obtaining the related literature. This involves collecting a wide range of resources, totaling 317 sources based on the keywords of this research, to ensure that this review has been conducted at a reasonable level. Then, the collected literature is organized and refined. This includes classifying the studies, summarizing the findings, and determining which studies are most relevant to the research objectives. The final stage focuses on evaluating the quality and relevance of the literature and reporting the findings. Ultimately, 110 articles were used as references in this research. It includes analysis of results, case studies, conclusions, and presentation of information in a clear and structured manner. The aim of this study is to provide a strong foundation for understanding existing features and functions in biomimetic architecture, focusing on the potential of plants to inspire the application of nature’s knowledge in this field.

### Terminology Definitions

Researchers have been studying biomimicry for several decades. A contemporary understanding of biomimicry has been clarified through many topics. Therefore, illuminating the scope of exploration and the importance of common terms related to it in the classification of academic fields is crucial [61,68]. For this purpose, the most important words and phrases were presented in Table 2. These words were used in preparing this article, demonstrating the importance of studying biomimicry. This perspective emphasizes the interdisciplinary impact and expansion of biomimicry, creating an opportunity for specialists in various fields to collaborate and participate in discussions.

**Figure 3:**
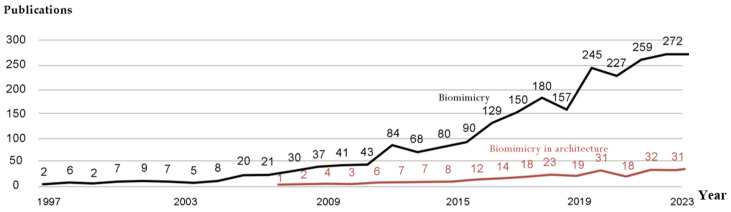
The total number of publications in Scopus from 1997 to 2023.

Furthermore, studies indicate that since the inception of this field in 1997 until 2024, there has been an increasing interest in biomimetic and biomimetic structures, and the esteem for this science is growing day by day. Figure 3 shows increased publication in biomimicry and biomimetic architecture, which is an indication that this field is becoming more popular not only in architecture but in other sciences as well.

## 4. Analysis of Case Studies (Results)

This section analyzes and examines the five levels of biomimicry architecture (refer to Figure 2), focusing on natural solutions inspired by plants, in order to understand the effectiveness of this approach in contributing to the use of natural light for energy-efficient buildings.

### 4.1. Form

Forms and architectural shapes can be very effective in building lighting and benefiting from natural light. The shapes found in nature provide abundant solutions that allow designers to improve the use of daylight, both in the overall form and in the details of the building form, and control its entry into the interior space, drawing inspiration from them [30]. On the other hand, plants, due to their reliance on sunlight for growth and survival, have forms and shapes that can make the most of natural light. This dependence on natural light not only influences their physical appearance but also shapes their growth patterns and overall physiology [74].

**Figure 4:**
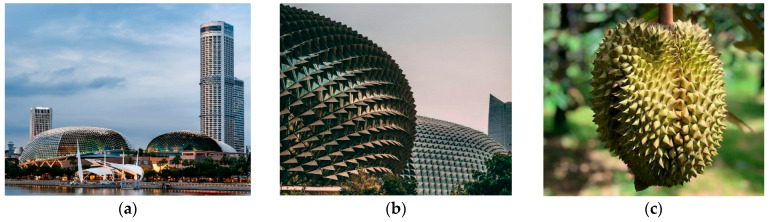
The Esplanade Theatre, Singapore. (**a**) Distant view of the building (**b**) Close-up view of the building (**c**) Durian as a symbolic fruit in Singapore.

Figure 4a,b shows the Esplanade Theatre, which is located in Marina Bay near the historic Singapore River. Architect Michael Wilford initially intended for the Marina Bay Splendid Theatre, a music hall and cultural venue, to have a transparent ceiling to provide a more accurate view from inside the building [75]. However, due to the constant heat and humidity of Singapore, a curved glass ceiling might cause the building to overheat. This led this project to draw inspiration from the durian tree in the area, a symbolic fruit (Figure 4c) of Singapore. The durian fruit is composed of three layers; the spongy middle layer has thermal properties that help preserve the durian fruit, while the outer layer has spiky features that protect the fruit from excessive heat from sunlight. The architect applies strategies of the double-layer shell by replacing the external triangular aluminum lamella fins, which are geometrically different and are calculated as a second skin above the glass dome based on the sun’s path throughout the year [73].

The design included a double-layered canopy with the second layer on top of the glass ceiling. This biomimetic design reduces the total energy consumption in the building by up to 30% and cuts down the use of artificial lighting by up to 55% [60,76]. The design utilizes a two-layer approach using triangular aluminum windows of different shapes that are determined annually by the sunlight paths on the second layer over the glass ceiling. To create maximum shade in all directions, aluminum windows are used throughout the building in such a way that their points are placed at different heights and angles depending on the locations of the daily sunlight paths annually [73,76].

The structure features a thermoplastic waterproofing membrane for single-layer waterproofing on the roof. This homogeneous elastic membrane contains a high number of solid materials with a high molecular weight that remains permanently stable over time. This consistent quality guarantees excellent durability, an optimal balance between tensile strength and elongation, as well as resistance to high temperatures and mechanical impact [77].

**Figure 5:**
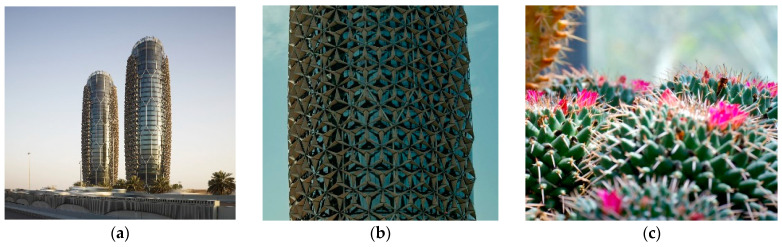
Al Bahr Towers, Abu Dhabi, United Arab Emirates. (**a**) Distant view of the building (**b**) Close-up view of the building (**c**) Cacti and its flowers as a source of inspiration.

In another example shown in Figure 5a,b, Al Bahr Towers’ integrated system is built on the concept of bio-inspired architecture, performance-oriented technology, and local architecture [78]. This design incorporates dynamic shading surfaces to meet sustainable development standards, utilizing natural daylight effectively and managing solar heat gain [79]. One of the goals of this design was to discover ideas inspired by biology. For this purpose, various natural systems were inspired by nature, from cacti and their flowers. Cacti have a canopy-like design that protects them from delicate skin, while their flowers open and close in response to weather changes (Figure 5c) [80]. This building, with its smart facade design, has improved user comfort and increased the physical and mental well-being of residents. It offers better spaces that naturally brighten up through improved natural light acceptance, advanced external views, reduced use of obstructive curtains, and enhanced comfort by reducing heavy air conditioning loads.

**Figure 6:**
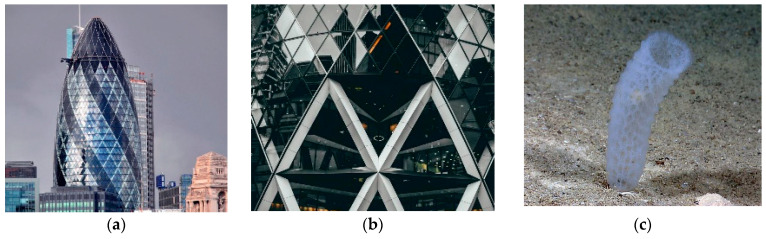
Swiss Re headquarters in London. (**a**) Distant view of the building (**b**) Close-up view of the building (**c**) Marine organism with several layers.

Figure 6a,b presents the Swiss Re headquarters in London. This building takes inspiration from a glass sponge and features a series of triangles on its exterior that resembles the shape of a sponge. This marine organism is made up of several layers of glass, and despite the small size of the threads, it creates a very sturdy skeleton (Figure 6c). This skeleton consists of networks of fibers that make up square cells, which are held together by other fibers that are arranged diagonally, thus describing spirals. The outer structure of the Swiss Re headquarters mimics the skeleton of Euplectella [60]. Its aerodynamic shape and glazing minimize wind loads while maximizing natural light and ventilation [15]. This form disperses reflected light and deflects gusting winds, thereby enhancing its environmental effects. The building structure was constructed in a grid form using the diagrid-architecture system inspired by the lattice-like exoskeleton of the Venus’ flower basket for a strong assembly of lightweight steel structure [73,81]. The empty spaces are stacked to form a spiral atrium space in the building. The spiral light well inside directs fresh air up through the building and greatly reduces energy consumption with a double-glazed curtain wall [82,83]. Ultimately, these design features have led to an 80% reduction in the building’s energy consumption [15].

### 4.2. Materials

Based on research conducted on plants and their mechanism, plants have a high potential to provide suitable solutions for improving building materials that meet human needs. For example, studies show that the porous internal structure of plant leaves and their biological composition of water and chlorophyll can create a spectral reflection effect on the leaves and produce materials resistant to sunlight [84]. Additionally, mimicking plants with surfaces containing defects, pores, and veins can help create semi-transparent assisting materials [80]. Plants with very fine veins on their leaves not only prevent water from sliding on their surfaces but also can be effective in reflecting sunlight and be compatible with warm and dry climates [85]. On the other hand, the tensile and flexible structure present in some plants can assist in creating suitable tensile materials in construction [53].

**Figure 7:**
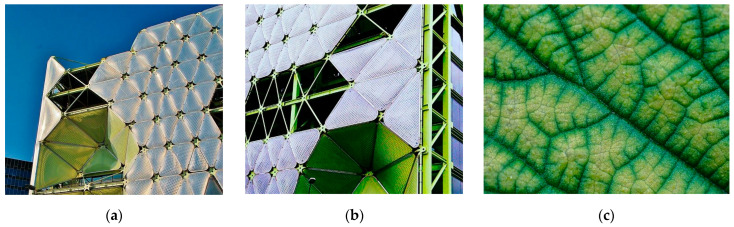
Building façade of Media-TIC building, Barcelona, Spain. (**a**) Distant view of the building (**b**) Close-up view of the building (**c**) The porous internal structure of plant leaves.

As shown in Figure 7a,b the implementation of a responsive facade system in a building envelope has been considered in the Media-TIC building. Figure 7c shows how the system can control the solar heat gain and improve the indoor environmental quality. ETFE is a hybrid material (Ethylene Tetra Fluoro Ethylene) with special characteristics. ETFE enhances insulation and light quality, while sensors adjust the layer’s transparency according to sunlight, managing heat gain and improving indoor environmental conditions. The cushion covering system is constructed using ETFE polymer and is enclosed with lamella fins. The pneumatic mechanisms of the lamella fins are automatically activated by light sensors inspired by the plant’s response to sunlight, which provides an active response to solar energy occurrence and improves thermal insulation [86]. This significantly lowers the solar factor of the building four times and reduces energy consumption by about 20% [80,85]. The mechanism used in this building was developed after comprehensive research, showing a very low economic cost compared to the project, making up 5% of the total [87].

**Figure 8:**
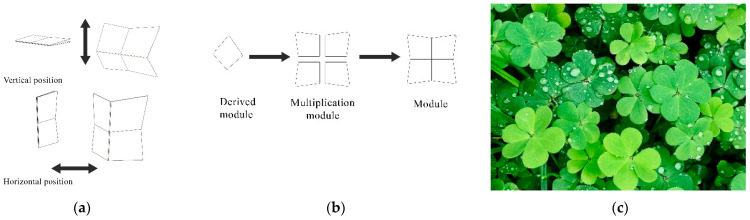
Application of vertical foldable shading devices on an office building, Lahore, Pakistan. (**a**) Shadow devices in horizontal and vertical axes (**b**) Module procedure (**c**) Agave leaf mountain mint.

In another design shown in Figure 8a, agave leaf mountain mint (Figure 8c) was considered as a primary concept for a 20-story office building in Lahore, Pakistan. Designing an adaptive bioclimatic façade as a practical solution to increase energy efficiency in high-gloss buildings in hot and humid areas has been considered [88]. The main facade module consists of four shadow devices that can tilt in both horizontal and vertical axes. In addition, this facade is able to generate its own electricity in addition to solar shading [89]. An adaptive biomimetic facade with adjustable shading devices that operate in horizontal and vertical positions effectively manages solar gain. As shown in Figure 8b, the shading facade, inspired by this plant, provides solar control without obstructing the user’s view. This building has succeeded in reducing energy consumption by 32% and decreasing illuminance levels (500–750 lux) to 50% of the total area [90].

### 4.3. Structure

The structure present in plants always provides suitable solutions to create a more stable and suitable structure. Based on studies in this area, we can leverage the ratios and structures found in plants to ensure that sufficient light reaches internal points while controlling the incoming light [44,91]. Furthermore, the structure of branches branching off tree trunks can not only help in creating columns with higher load-bearing capacity but also lead to better light absorption in indoor spaces [13].

**Figure 9:**
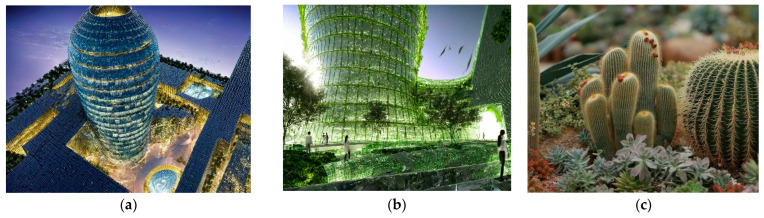
Shading system in Cactus Tower, Doha, Qatar. (**a**) Distant view of the building (**b**) Close-up view of the building (**c**) Cactus plant.

As shown in Figure 9a,b, the Cactus Tower was designed for the Ministry of Urban and Agricultural Affairs in Doha, Qatar, which was inspired by the cactus plant (Figure 9c). This building has a shading system that is visible on its facade, with building panels resembling cactus spines drawn on the building’s facade to create shade and regulate light entry. They can automatically open and close, oscillate, and adjust the amount of sunlight and heat transferred to the space to regulate the internal temperature as desired [81,92]. This solution allows this building to lower the size and amount of artificial cooling necessary for the building to 5–10%, so about 40–45% of the annual cost can be deducted, and also, the energy consumed by the HVAC can be decreased to 50% [92].

**Figure 10:**
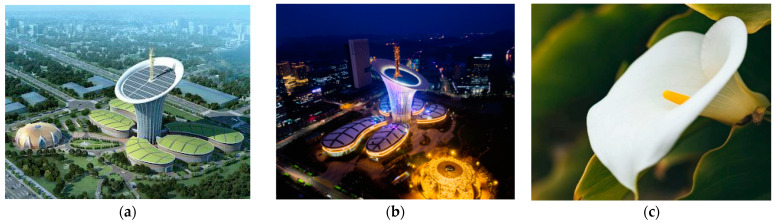
Natural light penetration in Wuhan New Energy Center, China. (**a**) Distant view of the building (**b**) Arial view of the building at the night (**c**) Lily flower.

In another project, a lily flower (Figure 10c) was used to inspire the Wuhan New Energy Center in China (Figure 10a,b). This building was designed based on a structure that meets its needs through renewable energies, both in terms of form and structure. A 140 m tower is surrounded by shorter towers resembling blossoms. The inner peak extends upward in a bowl-like structure, topped by a large sun-oriented exhibition space. Much like a real plant, it effectively absorbs sunlight [93]. By collecting and recycling rainwater and using solar panels, this building presents a suitable performance for a sustainable future [94]. The building has an aluminum pipe system that not only provides natural ventilation but also brings light to indoor spaces without natural light in the building. Additionally, appropriate shading for the building facade has been used for light modulation and shadowing. The building is also designed to maximize the absorption of sunlight and obtain the necessary energy for water heating and electricity. Based on reports and studies conducted, this building has been able to reduce about 91% of its energy consumption compared to conventional buildings [95].

### 4.4. Process

All plants have their own specific and complex processes that inspire architects. Biological processes such as growth, evolution, and reproduction in plants are inspiring architecture. For example, by taking inspiration from the growth process of plants, self-replicating structures can be created in buildings [96]. Plants use their exceptional capacity to harness energy from sunlight through the process of photosynthesis in nanomimic [97]. Cyanobacteria, green algae, seaweed, and some other plants have photosynthetic organisms capable of converting solar energy into electricity.

**Figure 11:**
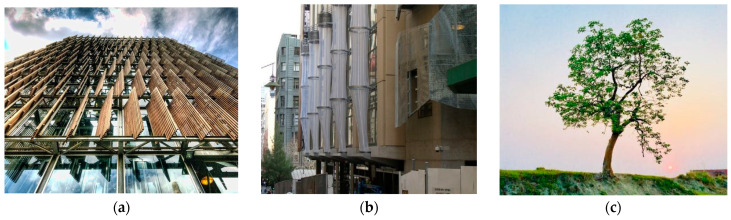
Council House 2 (CH2), Melbourne, Australia. (**a**) Distant view of the building (**b**) Close-up view of the building (**c**) Bark tree.

As shown in Figure 11a,b, Council House 2 (CH2) is a 10-story sustainable building situated in Melbourne, Australia. The building’s design was groundbreaking and inspired by emulating tree bark (Figure 11c). Throughout the structure, biomimicry was incorporated, with the west facade resembling the tree’s epidermis to regulate the external climate [1]. The north and south facades took inspiration from the tree’s bronchi, acting as a protective layer that filters light and air into the ventilated wet areas. This façade is covered with timber louvers to optimize the penetration of natural lights and views [29]. CH2 boasts a rooftop garden, encouraging biodiversity and reducing the urban heat island.

### 4.5. Function

Plant performance mimicry is an important aspect in this field because the performance of structures is very important, and the more suitable the performance, the more efficient the structure will be. Therefore, utilizing plant performance, such as mimicking the sunflower rotation toward the sun, can be effective in storing natural light and improving the efficiency of solar panels [55,98]. Additionally, by creating the ability to open and close window coverings in a building inspired by the mangrove flower, one can significantly impact the building’s performance in controlling daylight [99]. Moreover, controlling light from high surfaces like the plant *Fenestraria rhopalophylla*, which absorbs light through a window in its top [85], can also be utilized for shading purposes using the phyllotactic geometry present in plants to minimize self-shading of the building [53].

**Figure 12:**
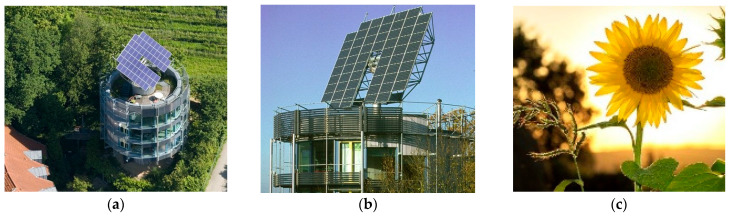
The Rotatable Solar, Germany. (**a**) Distant view of the building (**b**) Close-up view of the building (**c**) The artificial sunflower.

The Rotatable Solar House building in Germany shown in Figure 12a,b is inspired by the mechanism of the sunflower and known as the artificial sunflower (Figure 12c); it places the sun at its center and rotates the building to achieve sufficient daylighting. It features several nano-scale light absorbers and solar panels that absorb and store natural light throughout the day [81,100].

This building follows the sun’s rotation to provide more clean energy and adhere to environmental sustainability principles [101]. Plants like to adjust their orientation and preferences according to different weather conditions. Reorientation is driven by the preferences of leaves and differential growth in expanding leaves, especially occurring behind the leaf, leading to leaf curvature [57]. Essentially, the building, like a plant that adjusts its status relative to changing environmental conditions, reacts to what is happening around it [102]. Additionally, the building’s tracking is controlled by a computer system and can be adjusted to desired conditions and angles. The overall form of the building can be likened to a solitary tree that prevents heat loss with appropriate triple walls [100,103]. The adaptive shading of this façade provides a total energy saving of up to 82% and reduces the use of artificial lighting by 65% [104].

**Figure 13:**
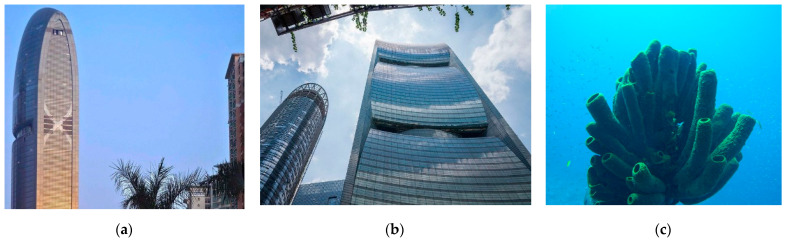
Pearl River Tower, China. (**a**) Distant view of the building (**b**) Close-up view of the building (**c**) Sea sponges.

As shown in Figure 13a,b, in another project, Pearl River is a 71-story tower designed by Skidmore, Owings, and Merrill that was completed in 2011. The building is renowned for being the first so-called ‘zero energy’ building, as it was designed to produce as much energy as it consumes [82]. The architects took inspiration from sea sponges (Figure 13c), creating a sponge-like structure and using this idea to reduce energy consumption in their designs [105]. They modeled their designs after the sponge’s ability to absorb gallons of water and organisms daily to reduce energy consumption. This porous tower features four openings, each housing a wind turbine, including one in the Pearl River Tower that generates electricity from strong winds. Additionally, the building harnesses solar energy through its photovoltaic system, integrated with the external solar shading and glass facade. The external solar shading system of the building and its glass facade, along with other energy-saving measures like radiant cooling, reduce the building’s energy consumption by 58–60% [106].

### 4.6. Case Studies Evaluation

Based on the study results and analysis of case studies inspired by biomimicry to optimize energy consumption through natural light, plants demonstrate significant potential for modeling solar energy utilization patterns. This underscores the importance of further exploration at each of the five levels of biomimetic architecture. These case studies are analyzed and compared across the five levels to identify which offers the greatest potential for aiding architects and designers in creating environmentally efficient buildings and reducing carbon footprints.

**Figure 14:**
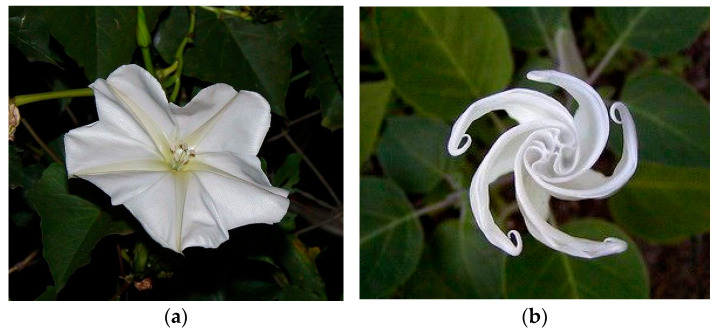
The movement of the moonflower plant. (**a**) extended position (**b**) folded position.

As the effectiveness of biomimicry in optimizing energy consumption depends on the specific conditions of each project, a universal guideline for drawing inspiration from plants in sustainable building design cannot be established. However, the findings aim to provide a general conclusion on the efficacy of these levels in utilizing natural light based on the case studies and research conducted. Table 3 summarizes the above case studies and their inspiration sources, and Figure 14 illustrates the energy conservation in each of the investigated buildings, which ranges from 20% to 90%.

The process and performance levels in biomimetic architecture often play a more pivotal role in reducing energy consumption through the utilization of light and solar energy. These levels were applied to various aspects of energy and sunlight management, such as light transmission, shading, and accounting for the sun’s path, resulting in higher energy preservation compared to other levels.

Following these, the structural level demonstrated considerable potential for energy preservation by mimicking plant structures. This approach effectively aids buildings in utilizing natural light, contributing to a reduced carbon footprint. At the final levels, form and materials are discussed. These have the least impact on reducing energy consumption through light control and utilization during the day, as observed in the case studies. Additionally, specific forms and materials can pose greater challenges due to higher construction costs and the need for specialized labor, making them less practical compared to the other levels.

## 5. Discussion

By drawing inspiration from nature, biomimetic strategies offer innovative solutions for energy efficiency, CO_2_ reduction, and climate resilience, addressing critical environmental challenges. The integration of adaptive materials, self-regulating building systems, and responsive façades can lead to more resource-efficient and low-impact construction methods. Furthermore, as climate change continues to shape building performance requirements, biomimicry provides a framework for creating resilient, self-sustaining structures that optimize natural resources like light, wind, and thermal energy. This study serves as a foundation for further research on scalable biomimetic technologies, interdisciplinary applications, and their role in shaping the future of regenerative architecture.

### Innovative Design Based on Findings

Based on the findings from the various studies conducted on the specific cases highlighted in this article, it becomes evident that nature, particularly the characteristics and functions of plants, serves as an excellent model and source of inspiration for designing buildings that are energy-efficient and maximize the use of natural light. In light of this understanding, this section will delve into a range of innovative solutions that draw from the principles observed in plants, demonstrating how these natural designs can be effectively implemented to achieve optimal utilization of natural light within the built environment. By examining these plant-inspired strategies, this study aims to illustrate the potential benefits that they can bring to modern architecture in terms of sustainability and energy efficiency. Artificial lighting, as the third consumer of energy in buildings, can be controlled through optimal daylighting design through the cost-effective biomimicry architecture concepts.

The effective utilization of sunlight in architectural design hinges on the careful management of the incoming light that permeates interior spaces. It is of utmost importance that buildings are conceived in a manner that not only mitigates the risk of excessive sunlight flooding the interior but also guarantees that the inhabitants benefit from sufficient natural light. This design approach must be balanced with the need to preserve unobstructed views of the outside environment for the users. Thus, this study proposal centers around the innovative concept of a movable facade that acts as a secondary skin for the building, effectively regulating the amount of light that enters the interior spaces. This unique facade design draws inspiration from the moonflower plant, known scientifically as Ipomoea alba, which exhibits remarkable characteristics that align with design goals.

As shown in Figure 14, *Ipomoea alba* L., known as moon vine, is a night-blooming morning glory native to tropical Americas. This plant is native to the American tropics but has naturalized in many places where it was introduced. The name moonflower derives from their blooming in the evening and their being round in shape like a full moon. The flowers open at night and are up to 6 inches (15 cm) across with a slender, equally long floral tube. The flowers have five sepals, five broadly rounded corolla lobes, and a pale greenish–yellow five-pointed “star” radiating from the center of the floral tube. Even though they are night-blooming, the large, shining white flowers are easy to spot if there is any moonlight. Leaves are green, heart-shaped to slightly lobed, and stems are twining [107].

**Figure 15:**
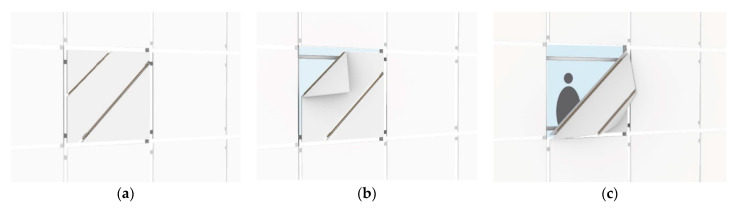
Proposed design for a double-skin facade. (**a**) Applicability of folding in two distinct positions (**b**) Folded in a time (**c**) Folded in two times.

The moonflower plant is particularly notable for its ability to change shape in response to the opening and closing of its petals. This attractive feature served as a key source of inspiration for the design proposed by this study, allowing for the creation of a movable facade that is not only flexible but can be folded into two distinct positions, as shown in Figure 15a. In the first case (Figure 15b), the facade is folded once, which significantly increases the brightness inside the space and creates a bright and attractive space. In the second case (Figure 15c), the facade folds twice, increasing the amount of light entering the building, which is especially beneficial during periods when natural light is at a minimum. Moreover, this adaptability of design may provide a healthier indoor environment, whereas wind is able to freely enter the building. The folding mechanism of this innovative building facade can be designed to operate either manually or mechanically and is equipped with light-sensitive sensors. In automatic mode, the facade would open and close based on the amount of light detected by the sensors, while in manual mode, users can adjust it according to their needs by disabling the mechanical function. Additionally, the dimensions of the facades can be customized according to the specific requirements of each unit in the building. This customization allows users to adjust the facade based on their individual needs and enables them to open and close it at will to optimize their experience of natural light and the external view.

Existing and constructed buildings can significantly benefit from improvements through proper design, focusing on several key areas. By emphasizing effective design strategies and focusing on a number of essential key areas, these structures can be enhanced in ways that significantly enhance their overall performance and aesthetic appeal [108]. One of the effective solutions in this regard is the improvement of the materials used in each organization. By utilizing contemporary materials that provide enhanced performance capabilities, it is possible to significantly improve the overall durability and resilience of buildings. These advanced materials can withstand various environmental stresses and contribute to a structure’s longevity and stability over time, ultimately leading to more robust construction outcomes [109].

Building shell designs inspired by the functionality of plant stomata present innovative solutions to some pressing challenges in architecture, particularly regarding energy efficiency and environmental management. Architectural shells with stomatal functions could benefit from biomimicry to better switch energy efficiency and environmental adaptability. Using those designs, the stomata opening and closing mechanism can be applied to modify shape depending on the light exposure and air quality in real time, enhancing aesthetics and functionality synergy [80,110].

**Figure 16:**
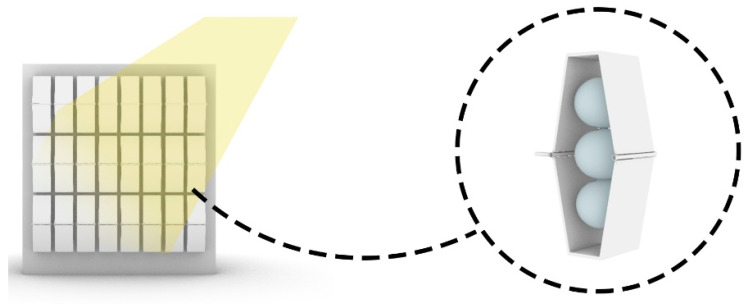
The shell in extended state in strong light conditions.

As shown in Figure 16, a solution for controlling incoming light through the façade, inspired by plant apertures and pneumatic systems, involves creating a secondary skin for the building with panels containing pneumatic cushions. This system expands with increased radiation and temperature, extending the façade to block sunlight from entering.

**Figure 17:**
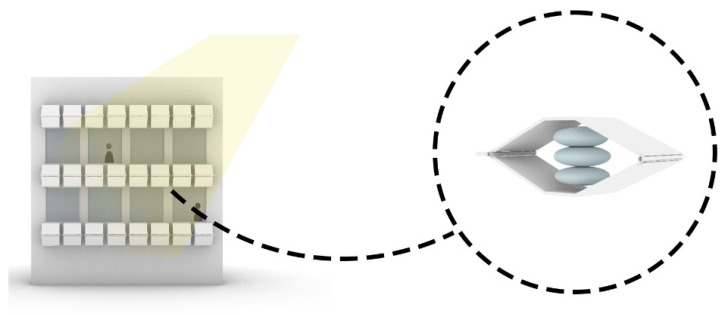
The shell is closed and in low light conditions.

Conversely, when radiation decreases and temperatures drop, the structure contracts, allowing natural light to enter through the windows, as shown in Figure 17. This dynamic process effectively manages incoming light, offering precise control reminiscent of natural shading mechanisms. Ultimately, this system enhances indoor quality and optimizes energy consumption in the building.

Based on studies conducted on the selected case studies in this article, it was found that by drawing inspiration from nature, and again looking at the natural patterns found in the world, we can propose suggestions to improve the performance of these buildings in order to reduce energy consumption and carbon footprint. One of the buildings where strategies can be implemented to improve its performance in terms of energy consumption is the Al Bahr Tower. This building has a dynamic façade that helps control the intense sunlight in the area, and it can enhance its all-glass exterior with a second movable shell that opens and closes based on the sun’s path. In the proposed solution, it is strongly recommended that the innovative second skin be implemented, such as this was effectively utilized in the facade of the Media-TIC building. This particular facade design incorporates semi-transparent materials that are specifically engineered to allow natural light to penetrate more deeply and evenly into the interior space. By doing so, it provides a filtered and refined quality of light that is ultimately more suitable for the interior environment. Additionally, this design approach plays a crucial role in minimizing heat gain, thus contributing to a more temperate and comfortable atmosphere within the building.

**Figure 18:**
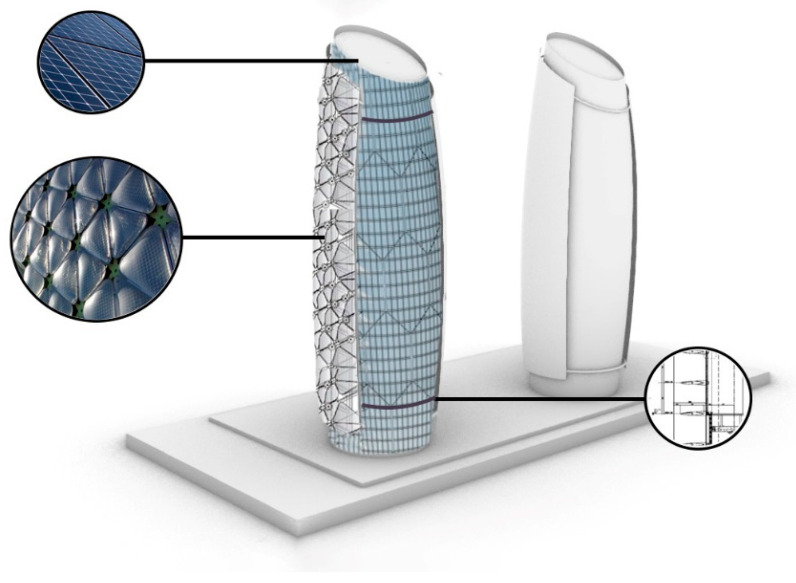
Proposed design combining reviewed case studies.

As shown in Figure 18, in addition to the innovative design, rails can be installed all around the perimeter of the building, enabling the proposed facade to freely move along these tracks, resembling a sunflower that elegantly tracks the path of the sun throughout the day. This dynamic movement will allow residents to benefit from increased exposure to natural light during both low-light conditions and more suitable times of day, ensuring they have ample opportunities to enjoy the brightness that sunlight brings while also providing them with unobstructed views of the outdoor environment. Furthermore, akin to the energy-efficient model employed at the Wuhan Energy Center, it is also feasible to incorporate solar panels on the roof of this building. These solar panels would generate the necessary electricity to power this movable facade system as well as other essential operational needs of the building. By harnessing solar energy in this manner, we can significantly reduce overall energy consumption, contributing to a more sustainable and environmentally friendly approach to urban living. Finally, Table 4 presents suggested materials and structures, biomimicry level, inspiration and purpose of design for all proposed designs. These findings help designers to evaluate the efficiency of these applicable strategies in a real buildings.

## 6. Conclusions

This article offers a comprehensive review and evaluation of biomimicry inspired by plants, focusing on building form, façade interventions, function, structure, materials, and energy. Plants have a high potential for mimicking patterns of natural light, making their use in biomimetic architecture to optimize natural lighting a sustainable and innovative solution for creating healthier and more efficient built environments. It provides valuable insights into various practical applications of this approach, emphasizing distinctive design cases and outlining future trends in this area. Additionally, this article examines key points and case studies of biomimetic applications in architecture, demonstrating capabilities for energy savings and consumption reduction in buildings. Case studies have shown how mimicking patterns, forms, and systems in nature can lead to more sustainable, efficient, and aesthetically pleasing spaces. A comprehensive review of the relevant literature from 2005 to 2024 revealed that despite numerous studies and designs in the field of biomimetic architecture, there is significant untapped potential for advancing this approach, necessitating further research in this direction. The efficiency of utilizing renewable energy sources indicates that the development of biomimicry technologies for building performance should be prioritized since this approach is critical for designing environmentally friendly buildings. The balanced and mutually beneficial relationship between green buildings and the environment, while reducing the environmental impacts of structures, is one of the important goals and advantages of biomimetic architecture. To create green structures inspired by plant biomimicry, adherence to the principles of nature and their integration with building technology, considering a comprehensive understanding of the relevant biological processes of plants is essential. By simulating the growth structure and environmental performance of plants, a healthy ecosystem can be formed to foster the development of energy-efficient bionic buildings and green structures. In addition, this study proposed theoretical solutions for future developments based on the review findings. However, further investigation into these proposals is necessary to refine, validate, and implement them effectively, ensuring their practical feasibility and maximizing their impact on advancing biomimetic architecture.

## Figures and Tables

**Table 1:** Literature review design.

Study Stage	Identification
First	Review domain: biomimicry architecture, natural light, plants. Review questions: How much research has been conducted in this field? Which are the leading countries in this field of research? Source type: journal articles
	Acquisition
	Search mechanism and material acquisition: Scopus, Science Direct, and Google Scholar database Search Period: 2005–2024 Keywords: biomimicry architecture, biomimicry design, biomimicry and sustainability, use of natural light in biomimicry architecture, light simulation, biomimicry and plants, case study of biomimicry design.
Second	Organization
	Organization Codes: Publication Trend Analysis, Keyword Co-Occurrence Analysis, Co-Authorship Analysis. Organization Frameworks: challenges and potentials of the desired approach
	Purification
	Article Type Excluded: proceeding and book, editorial Article Type Included: 120 articles for analysis (journal articles, review articles)
Third	Evaluation
	Analysis Methods: bibliometrics, analysis, and review of case examples Agenda Proposal Method: thematic analysis.
	Reporting
	Reporting Conventions: figures, tables, networks, graphs, and words. Limitations: Data accessed from dimension review are limited to bibliometric data
Overview of SPAR-4-SLR Protocol Framework (Source: Adapted from JOB Rotimi [67])	

**Table 2:** Key terminology of research keywords and definitions.

Keyword	Definition
Bionic	Bionics is a term invented to describe the prospective field, involving copying, imitating, and learning from nature [9].
Bio-morphology	It means creating structures that resemble natural organisms, meaning that the buildings have skin similar to natural shapes and are only inspired by nature in terms of appearance [31].
Phyto-mimetics	When inspiration is drawn from the structure and functionality of plants, it may be referred to as phyto-mimetics [43].
Biomimicry	Biomimicry is an approach to innovation that seeks sustainable solutions to human challenges by emulating nature’s time-tested patterns and strategies [53,69].
Biomimetic	This word is made up of parts including “bio” (life) and “mimesis” (imitation). Biomicism can be defined as forms inspired by nature, adaptation, or application in architecture, which can be examined in two ways: replication of natural objects and formation process in constructions [70,71].
Biomechanic	Biomechanics is a scientific field that applies biological and engineering principles to living organisms. It studies organism movement, control of those movements, force impact, and pressure on tissues [72].
Bio-inspiration	This concept focuses on using nature for inspiration in design without copying it directly. Bio-inspiration uses nature for ideas and patterns to find creative solutions [73]. Mimicking shapes employs technology to create sustainable materials similar to natural objects [10].

**Table 3:** Application of plants for optimizing natural light.

Case	Biomimicry Level	Inspiration	Biomimicry Concept	Problem Solved	Energy Efficiency
Esplanade Theatres	Form, Material	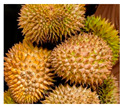	Second layer of protection for underlying layers	- Controlling input heat - Shading	30%
Al Bahr Towers	Form, Function	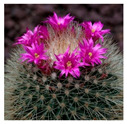	A movement that controls the amount of natural light coming in and makes good use of it Daylight control	- Controlling the entry light and heat - Moving shading	50%
Swiss Reinsurance Headquarters	Form, Structure	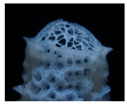	Use a series of outer triangles to create the shape of a glass sponge.	- Light control - Ventilation	80%
Media-TIC	Structure, Material	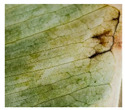	Transparent building skin insulation and light-sensitive, responsive to intense light	- Enhance light quality - Light control - Heat control	20%
Lahore office building	Material, Form	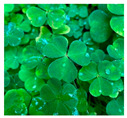	Designing a facade shadow pattern inspired by the physical structure of a plant leaf	- Visual comfort - Reduction in solar heat - Energy consumption reduction	32%
Qatar Cactus Tower	Form, Function	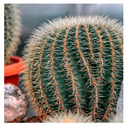	Reduction in heat and intense light penetration	- Adjusting the input light - Adjusting internal temperature	50%
Wuhan New Energy	Form, Structure	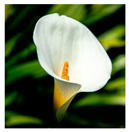	Transfer and storage of natural light	- Light transmission - Solar energy storage	91%
Council House 2	Process, Function, Material	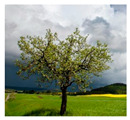	It filters the light and air before entering the building.	- Controlling space light - Solar energy storage	82%
Rotatable Solar House	Structure, Function	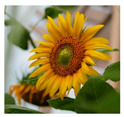	Maximizing the use of sunlight	- Light absorption - Solar energy storage	82%
Pearl River Tower	Form, Function	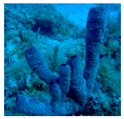	Inspired by the structure of sea sponges to generate electricity and control daylight	- Electricity generation - Reduce artificial light consumption - External shading	60%

**Table 4:** Potential structures of proposed designs based on study findings.

Proposed Designs	Suggested Material	Suggested Structure	Biomimicry Level	Inspiration	Purpose
Proposed design 1	PTFE	Double-skin façade, Metal support frames	Structure	Moonflower	Light control and energy consumption reduction
Proposed design 2	Composite Panel, ETFE	Double-skin façade, Pneumatic	Function, Material	Plant pore	Light control and energy consumption reduction
Proposed design 3	ETFE	Pneumatic	Function, Structure	Sunflower and cactus	Utilizing solar energy and reducing energy consumption

## Data Availability

The data presented in this study are available upon request from the corresponding authors. The data are not publicly available due to confidentiality.
